# Preferences for Shared Language for Health Equity Across the Political Spectrum

**DOI:** 10.1001/jamanetworkopen.2026.0277

**Published:** 2026-03-06

**Authors:** Samantha X. Y. Wang, Sujin Song, Margaret C. Nikolov, Zakary Tormala, Robert M. Kaplan, Kevin Schulman

**Affiliations:** 1 Division of Hospital Medicine, Department of Medicine, Stanford School of Medicine, Stanford, California; 2 Clinical Excellence Research Center, Stanford School of Medicine, Stanford, California; 3 Stanford Graduate School of Business, Stanford University, Stanford, California; 4 Division of Primary Care and Population Health, Department of Medicine, Stanford School of Medicine, Stanford, California

## Abstract

**Importance:**

The language used to describe health equity efforts has become increasingly contested. Understanding how language influences public attitudes is essential to effectively communicate equity-focused concepts across ideological groups.

**Objective:**

To assess the association of language and framing with public receptiveness to health equity concepts, including alignment of definitions with shared values, reactions to common terms, and preferences for public health framing statements.

**Design, Setting, and Participants:**

This cross-sectional online survey of 1000 US adults was conducted between April 9 and April 25, 2025, using an international public opinion research firm that recruited from a nonprobability-based online panel with matching and weighting to national benchmarks. Participants were stratified by age, sex, race and ethnicity, self-reported political ideology, and geographic region to approximate national demographics.

**Exposures:**

The survey included 3 modules: (1) random assignment to a definition of *health equity* or *health equality* with values-alignment questions; (2) evaluative reactions to 10 common health equity terms; and (3) preferences for 4 pairs of equivalent public health statements differing only in framing.

**Main Outcomes and Measures:**

Primary outcomes included participant ratings of values alignment (including personal values and values core to national identity, the latter of which were termed *American values* in the survey), evaluative reactions to equity-related terms (negative to nonnegative), and framing preferences across ideology groups. Descriptive statistics assessed values alignment, reactions to terms, and framing preferences.

**Results:**

Among 1000 US adults (weighted number, 513.2 [51.3%] female; 105.8 [10.6%] very liberal, 164.0 [16.4%] liberal, 344.0 [34.4%] moderate, 205.9 [20.6%] conservative, 98.7 [9.9%] very conservative, and 81.5 [8.2%] not sure), respondents represented a broad range of ages, racial and ethnic backgrounds, and educational attainment. Respondents assigned to the health equity definition reported higher alignment with personal values (42.9%-87.4% across groups) than those assigned to health equality (28.5%-79.0% across groups), with endorsement of either concept increasing progressively from very conservative to very liberal respondents (*P* < .001 in both cases). When assessing perceived core American values, health equity (47.0% [95% CI, 33.1%-60.8%]) was more frequently endorsed than health equality (21.1% [95% CI, 9.6%-32.5%]) among those identifying as very conservative. Four terms—*accessible health care*, *health care investment*, *population health*, and *community health*—were broadly well received (<10% negative responses in most groups), whereas the terms *marginalized communities* and *inclusive health* elicited more divergent reactions across ideologies (*P* < .01 in both cases). Collectivist (*our health* vs *your health*) and affirming (*start*, *support*, and *increase*) framings were consistently preferred across groups.

**Conclusions and Relevance:**

In this national online survey of 1000 US adults, respondents showed both ideological differences and areas of convergence in responses to health equity language and framing. These findings suggest that strategic use of broadly resonant terms and collective, affirming framings may foster understanding of and support for health initiatives across political ideologies.

## Key Points

**Question:**

Are differences in language and framing associated with public receptiveness to health equity concepts across political ideology groups?

**Findings:**

In this survey study including 1000 US adults, a higher proportion of respondents endorsed personal values alignment with *health equity* than *health equality* definitions. The terms *marginalized communities* and *inclusive health care* elicited divergent reactions, whereas *accessible health care*, *health care investment*, *population health*, and *community health* were broadly well received; across all groups, collectivist, affirming, action-oriented statement framings (eg, *we*, *start*, *support*, *increase*) were preferred.

**Meaning:**

These findings suggest that strategic use of language emphasizing shared values and collective action may increase public understanding and receptiveness to health equity initiatives.

## Teaser

This survey study of US adults explores the alignment of *health equity* or *health equality* definitions with shared values, assesses reactions to commonly used health equity terms, and identifies framing preferences between paired public health statements about health equity.

## Introduction

Recent changes in federal policy have altered the landscape of public health programs, including reductions in funding for disease surveillance, prevention initiatives, community health infrastructure, and equity-focused efforts.^1,2,3,4^ These changes have occurred in the context of long-standing underinvestment in public health.^5^ These shifts have raised questions about how the public perceives current health equity efforts and whether communication strategies can bridge differences in understanding across ideological groups. Recent federal actions have challenged public health infrastructure and curtailed programs designed to reduce health disparities and advance population health.^6,7,8,9^ While these steps are a clear political response to public health initiatives, it is not clear if these actions represent the interests and values of the broader public.

Research has shown that both topic and context shape the effectiveness of health and science messaging and that audience values, including political ideology, influence how messages are received.^10^ This concept has been described as information framing.^11,12^ While no single framing approach resonates uniformly across the political spectrum, emerging evidence suggests that certain message attributes, such as emphasizing personal responsibility, economic stability, or national strength, may hold greater appeal for some, whereas others may respond more positively to frames invoking fairness, community care, or systemic change.^13,14,15^ Even small linguistic differences have been shown to affect public perception and policy support in areas ranging from the opioid crisis^16,17^ to childhood obesity,^18^ beverage regulation,^19,20^ and climate change.^14,15,16,19,20,21^

Strategic message framing has been proposed as a tool to strengthen public and legislative support for population health initiatives.^22^ However, as the discourse around equity-oriented programs has become increasingly contested, it remains unclear how specific health equity terms and concepts are received by the public. To examine how language influences receptiveness to health equity concepts, we conducted a national cross-sectional online survey of US adults in which participants assessed the alignment of *health equity* or *health equality* definitions with personal values and perceived national identity values (termed *American values* in the survey), reported reactions to commonly used health equity terms, and indicated their framing preferences by choosing between paired public health statements. We hypothesized that receptiveness to health equity language and framing would vary across political ideological groups, with some terms and messages eliciting broader acceptance than others.

## Methods

### Population Sample

We conducted this survey study in partnership with YouGov, an international public opinion research firm that recruits participants from a large, opt-in online panel and constructs samples to approximate national representativeness through matching and weighting procedures. Because the YouGov panel is nonprobability based and no pilot data were available to guide expected effect sizes, we did not perform an a priori power calculation. Instead, a target sample of 1000 respondents was selected because this size yields stable weighted estimates and is consistent with standard practice in national public opinion research for enabling subgroup comparisons.^23,24^ Political ideology was self-reported using standard YouGov response categories without additional prompts or definitions provided. Race and ethnicity were self-reported by participants using fixed categories provided in the survey instrument (Asian or Asian American, Black or African American, Hispanic or Latino, Middle Eastern, Native American [American Indian or Alaska Native], White, multiracial, or other [unspecified]), which were based on US Census classifications. Race and ethnicity were assessed because perceptions of health equity–related language may vary across social groups and differential experiences of health care in the US. Sampling frames were derived from US Census and election data, and respondents were matched to these frames on sex, age, race and ethnicity, and educational attainment using propensity score methods. Poststratification weights were applied to align with demographic and voting benchmarks. Data collection occurred between April 9 and April 25, 2025. The study was approved by the Stanford Institutional Review Board, and all participants provided electronic informed consent before beginning the survey. This study adheres to the American Association for Public Opinion Research (AAPOR) reporting guidelines for survey studies.

### Survey Instrument

Consistent with the AAPOR standards, we report the sampling frame, recruitment and matching procedures, weighting approach, survey administration details, and analytical methods. The survey instrument (eAppendix in Supplement 1) was developed by the coauthor team of social scientists with expertise in survey methodology and health communication (S.X.Y.W., Z.T., R.M.K., and K.S.), and through iterative review to optimize clarity and minimize ambiguity. The survey consisted of 3 modules. In the first module, Personal and Perceived American Values, respondents were randomly assigned (1:1) to view a definition of either *health equity* or *health equality*. To minimize confusion and potential carryover effects, respondents were randomized to respond to a single definition. After reading the assigned definition, participants rated the extent to which it aligned with their personal values and their perception of core American values on a 5-point Likert scale. Core American values were intentionally left undefined to capture the participant’s own interpretations. In the second module, Evaluative Reactions to Terms, participants rated their reactions to common terms used in health equity initiatives on a 7-point scale (−3, very negative; 0, neutral; +3, very positive). In the third module, Framing Preferences, participants reviewed 4 pairs of semantically equivalent public health statements that differed only in framing and selected their preferred framing or indicated no preference. The 4 pairs of statements included variations in *we* vs *you*, *support* vs *oppose*, *increase* vs *decrease*, and *start* vs *stop* framing.

Modules 2 and 3 were designed to be independent of module 1. They contained separate content and did not reference the assigned definition in module 1. Terms and framings were presented without reminders of earlier material. Thus, no carryover effects were expected.

### Statistical Analysis

To assess national representativeness, we compared the weighted demographic characteristics of the analytic sample with US population estimates from the American Community Survey, a nationally representative survey conducted by the US Census Bureau.^25^ Consistent with YouGov’s weighting approach, the correspondence across key demographic variables aligned closely with population benchmarks. These comparisons are provided in the eTable in Supplement 1. For module 1, we calculated the proportion of respondents agreeing or strongly agreeing that the assigned definition aligned with personal or American values. Because respondents viewed only 1 definition, differences reflect between-group comparisons rather than within-person preferences. For module 2, we evaluated the relative frequency of negative (−3, −2, or −1) vs nonnegative (0, +1, +2, or +3) reactions to 10 terms. For module 3, we examined preferences for each framing pair, including no preference. Both unweighted and weighted results were examined; results with and without weights were comparable, and weighted results are reported herein. The χ^2^ test of association was used to assess differences in agreement, reaction, and preference by ideology. One-sided *P* < .05 was considered statistically significant. Standard methods for proportions were used to construct (pointwise) 95% CIs for agreement (module 1), terms (module 2), and framing preference (module 3) by political ideology. Given the potential reduction in power when dichotomizing an ordinal measure (module 2), Kruskal-Wallis rank sum tests were conducted as well to assess ideological differences in reaction to terms. All analyses were conducted in R software, version 4.2.1 (R Project for Statistical Computing).

## Results

Respondents included 1000 US adults (weighted sample, 513.2 [51.3%] female and 486.8 [48.7%] male), ranging in age from 18 to 75 years or older, with racial and ethnic distributions representative of national composition (29.3 [2.9%] Asian or Asian American, 124.6 [12.5%] Black or African American, 105.1 [10.5%] Hispanic or Latino, 3.9 [0.4%] Middle Eastern, 18.2 [1.8%] Native American, 664.7 [66.5%] White, 29.0 [2.9%] multiracial, and 25.1 [2.5%] other), and political ideology distributed across very liberal (105.8 [10.6%]), liberal (164.0 [16.4%]), moderate (344.0 [34.4%]), conservative (205.9 [20.6%]), very conservative (98.7 [9.9%]), and not sure (81.5 [8.2%]). Additional demographic characteristics are provided in the Table. Missingness in survey responses was negligible, with just 2 respondents (self-described moderates) skipping a single survey item (term *accessible health care* in module 2).

**Table.** Characteristics of US General Population Survey Sample

Characteristic	Weighted No. (%)
Ideology: In general, how would you describe your own political viewpoint?	
Very liberal	105.8 (10.6)
Liberal	164.0 (16.4)
Moderate	344.0 (34.4)
Conservative	205.9 (20.6)
Very conservative	98.7 (9.9)
Not sure	81.5 (8.2)
Insurance (may be >1): Are you currently covered by any of the following types of health insurance or health coverage plans?	
Public (includes public plus private)	416.6 (41.7)
Private only	430.2 (43.0)
Other	36.1 (3.6)
Uninsured	114.4 (11.4)
General health condition: Would you say your health in general is excellent, very good, good, fair, or poor?	
Excellent	253.3 (25.3)
Very good	274.6 (27.4)
Good	309.0 (30.9)
Fair	129.0 (12.9)
Poor	34.0 (3.4)
Serious health events: In the past 12 mo, have you experienced any of the following serious health events?	
Hospitalization	97.7 (9.7)
Major surgery	59.2 (5.9)
Emergency care	55.0 (5.5)
Diagnosis serious condition	61.4 (6.1)
None of the above	811.4 (81.1)
Age range, y	
18-24	106.6 (10.7)
25-34	172.7 (17.2)
35-44	181.5 (18.2)
45-54	144.0 (14.4)
55-64	183.4 (18.3)
65-74	146.1 (14.6)
≥75	65.7 (6.6)
Sex: Are you…?	
Male	486.8 (48.7)
Female	513.2 (51.3)
Race and ethnicity: What racial or ethnic group best describes you?	
Asian or Asian American	29.3 (2.9)
Black or African American	124.6 (12.5)
Hispanic or Latino	105.1 (10.5)
Middle Eastern	3.9 (0.4)
Native American	18.2 (1.8)
White	664.7 (66.5)
Multiracial	29.0 (2.9)
Other (unspecified)	25.1 (2.5)
Hispanic ethnicity: Are you of Spanish, Latino, or Hispanic origin or descent?	
Yes	160.6 (16.1)
No	839.4 (83.9)
Marital status: What is your marital status?	
Married or partnered	525.9 (52.6)
Previously married	175.6 (17.6)
Never married	298.5 (29.9)
Educational attainment: What is the highest level of education you have completed?	
Did not graduate from high school	69.6 (7.0)
High school graduate	314.3 (31.4)
Some college or Associate’s degree	278.6 (27.9)
Bachelor’s degree	214.7 (21.5)
Postgraduate degree (MA, MBA, MD, JD, PhD, etc)	122.8 (12.3)
Employement: Which of the following best describes your current employment status?	
Employed	513.1 (51.3)
Unemployed	79.3 (7.9)
Student	51.8 (5.2)
Permanently disabled	61.9 (6.2)
Not in labor force	293.8 (29.4)

Abbreviations: JD, doctor of jurisprudence; MA, master of arts; MBA, master of business administration; MD, doctor of medicine; NA, not applicable; PhD, doctor of philosophy.

### Personal and Perceived American Values

**Figure 1.**
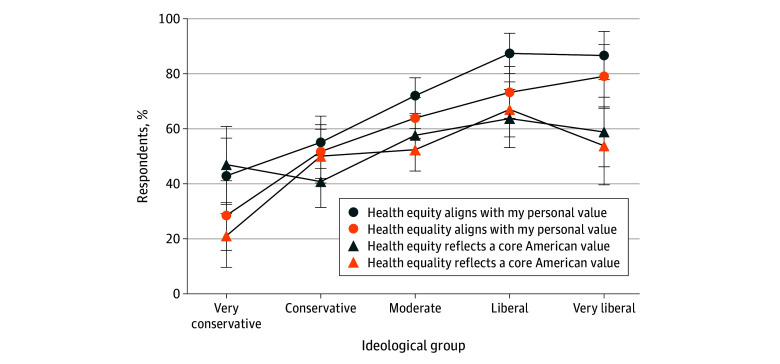
Line Graph Displaying Agreement With *Health Equity* or *Health Equality* Definition Across Ideological Groups Error bars indicate 95% CIs.

Module 1 assessed whether population health concepts aligned with personal values and perceived core American values. Agreement for definitions of *health equity* or *health equality* increased progressively across the political spectrum (Figure 1), from very conservative (42.9% [95% CI, 29.2%-56.6%] for *health equity*, 28.5% [95% CI, 15.8%-41.1%] for *health equality*) to very liberal respondents (86.6% [95% CI, 77.9%-95.4%] for *health equity*, 79.0% [95% CI, 67.5%-90.6%] for *health equality*), indicating an association between political ideology and alignment with personal values (*P* < .001 for both definitions). Across all groups, a higher proportion of respondents endorsed alignment with *health equity* than with *health equality*. When respondents were asked whether these concepts reflected a core American value, however, the results were more mixed. Among liberal and very liberal respondents, endorsement of *health equity* and *health equality* as core American values was similar (liberal respondents: 63.7% [95% CI, 53.1%-74.3%] vs 67.0% [95% CI, 57.0%-77.0%]; very liberal respondents: 58.8% [95% CI, 46.2%-71.4%] vs 53.8% [95% CI, 39.6%-68.0%]). In contrast, among very conservative respondents, a higher proportion assigned to the *health equity* definition endorsed alignment compared with those assigned to the *health equality* definition (47.0% [95% CI, 33.1%-60.8%] vs 21.1% [95% CI, 9.6%-32.5%]). Agreement that *health equity* and *health equality* are core American values differed across political ideology (*health equity*, 63.7% [95% CI, 53.1%-74.3%] liberal vs 47.0% [95% CI, 33.1%-60.8%] very conservative; *P* = .01; *health equality*, 67.0% [95% CI, 57.0%-77.0%] liberal vs 21.1% [95% CI, 9.6%-32.5%] very conservative; *P* < .001). The largest divergence between personal values and perceived American values was observed among liberal and very liberal respondents. In these groups, endorsement of both terms was substantially higher for personal values than for perceived American values.

### Evaluative Reactions to Common Terms

Reactions to health equity–related terms were first assessed on the original 7-point scale (range, −3 to +3). Kruskal-Wallis tests demonstrated significant differences in median reactions across political ideology for all terms except *vulnerable populations*. For interpretability, reactions were subsequently dichotomized as negative (<0) vs nonnegative (≥0).

**Figure 2.**
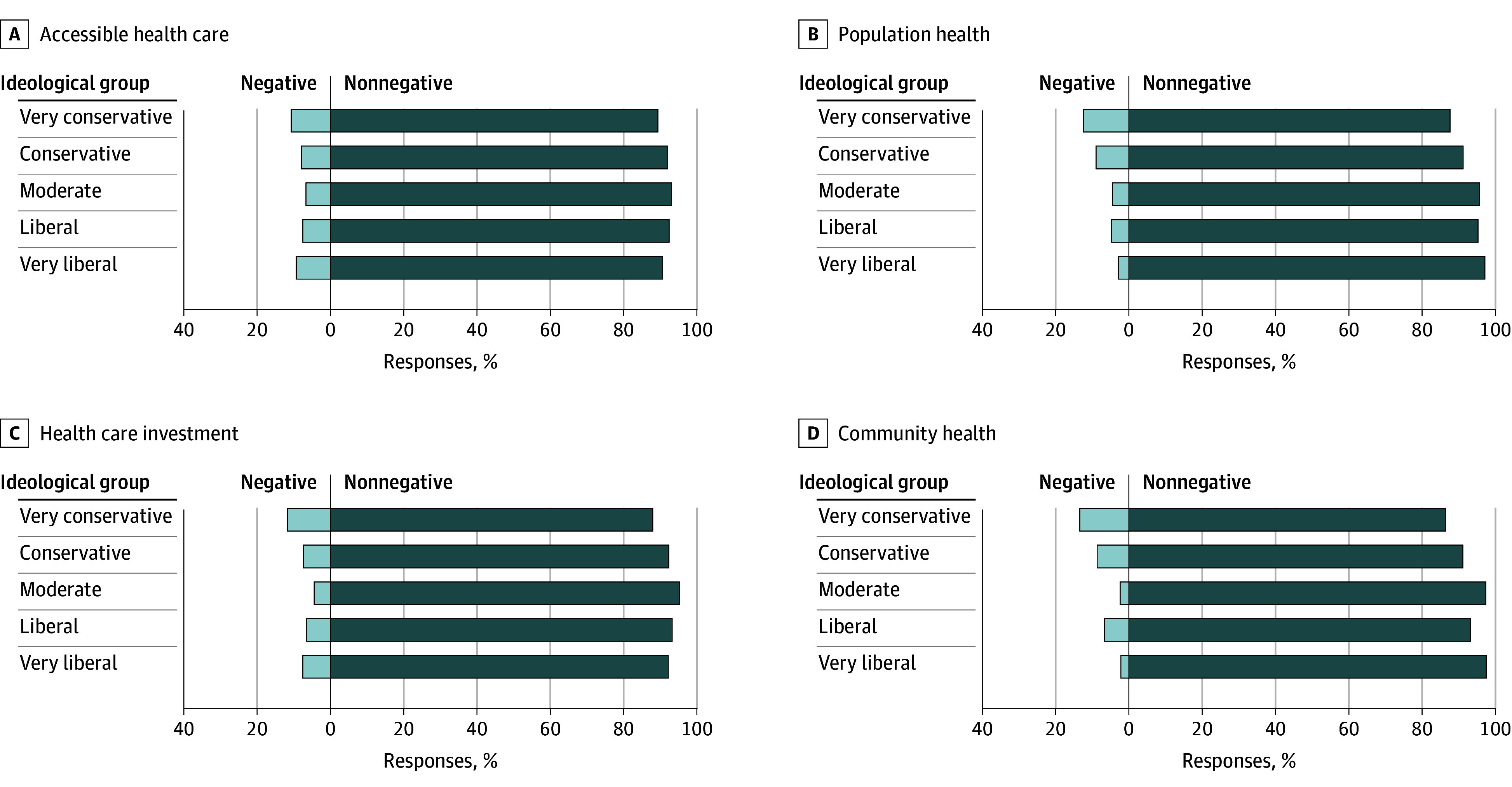
Bar Graphs Showing Common Health Equity Terms With Broadly Nonnegative Reactions Across Ideological Groups Four terms describing health equity were received favorably or neutrally: *accessible health care*, *population health*, *health care investment*, and* community health*. Most responses were nonnegative across ideological groups.

Across ideology groups, negative reactions were uncommon for *accessible health care*, *health care investment*, *population health*, and *community health*, with fewer than 10% of respondents expressing negative reactions in most groups (Figure 2). Although overall responses were largely neutral or positive, very conservative respondents expressed higher negative reactions to *population health* (12.3% [95% CI, 5.8%-18.8%] vs 8.9% [95% CI, 5.0%-12.8%] for conservative respondents to 2.8% [95% CI, 0-5.9%] for very liberal respondents; *P* = .008) and *community health* (13.5% [95% CI, 6.7%-20.2%] vs 8.6% [95% CI, 4.8%-12.5%] for conservative respondents to 2.2% [95% CI, 0-5.0%] for very liberal respondents; *P* < .001).

**Figure 3.**
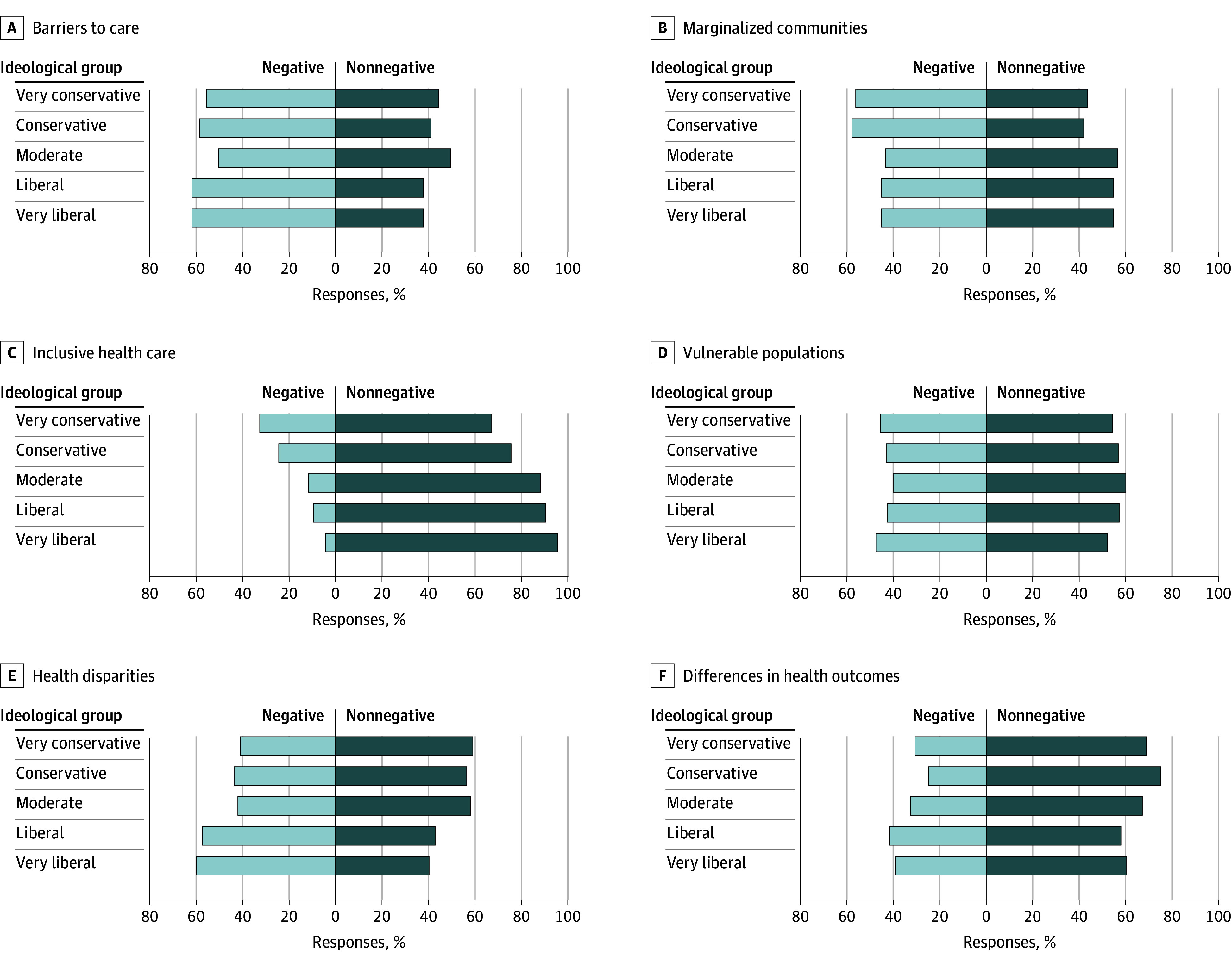
Bar Graph Showing Common Health Equity Terms With Mixed Reactions Across Ideological Groups Six terms elicited a mix of negative and nonnegative reactions. Reactions differed significantly by ideology in 4 terms, with more conservative and very conservative respondents expressing negativity toward the terms *marginalized communities* and *inclusive health care*, and more liberal and very liberal respondents expressing negativity toward the terms *health disparities* and *differences in health outcomes.*

Six other terms elicited more mixed reactions across ideology (Figure 3), with clear ideological divisions observed for *marginalized communities*, *inclusive health care*, *health disparities*, and *differences in health outcomes*. For *marginalized communities*, negative reactions were highest among very conservative (56.2% [95% CI, 46.4%-65.9%]) and conservative respondents (57.9% [95% CI, 51.1%-64.6%]), compared with moderate (43.3% [95% CI, 38.0%-48.5%]), liberal (45.1% [95% CI, 37.5%-52.7%]), and very liberal respondents (45.1% [95% CI, 35.6%-54.6%]) (*P* = .006). For *inclusive health care*, negative reactions declined progressively across the political spectrum, from 32.7% (95% CI, 23.4%-42.0%) among very conservative respondents to 24.4% (95% CI, 18.6%-30.3%) among conservative, 11.7% (95% CI, 8.3%-15.0%) among moderate, 9.5% (95% CI, 5.0%-14.0%) among liberal, and 4.3% (95% CI, 0.4%-8.2%) among very liberal respondents (*P* < .001). In contrast, negative reactions to *health disparities* were more common among very liberal (59.8% [95% CI, 50.5%-69.2%]) and liberal respondents (57.2% [95% CI, 49.6%-64.7%]) than among moderate (42.0% [95% CI, 36.8%-47.2%]), conservative (43.5% [95% CI, 36.8%-50.3%]), and very conservative respondents (41.0% [95% CI, 31.3%-50.7%]) (*P* = .001). A similar pattern was observed for *differences in health outcomes*, with higher negative reactions among very liberal (39.2% [95% CI, 29.9%-48.5%]) and liberal respondents (41.6% [95% CI, 34.1%-49.2%]) compared with moderate (32.6% [95% CI, 27.6%-37.6%]), conservative (24.8% [95% CI, 18.9%-30.7%]), and very conservative respondents (30.7% [95% CI, 21.6%-39.8%]) (*P* = .008).

### Framing Preferences for Public Health Statements

**Figure 4.**
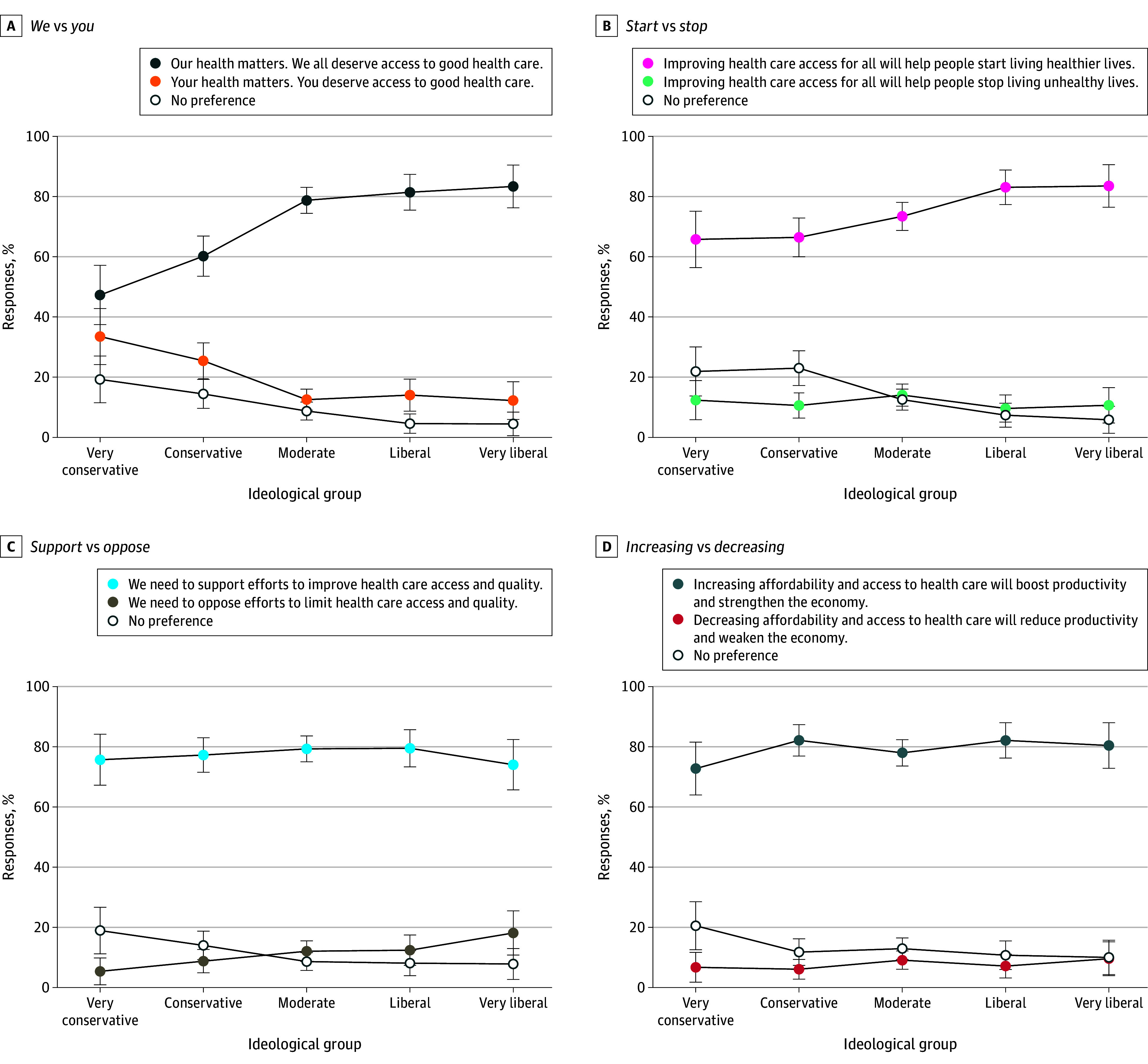
Line Graph of Preferred Framings of Public Health Statements Across Ideological Groups Error bars indicate 95% CIs.

Across ideologies, respondents preferred collectivist (*we*) over individual (*you*) framing, action-oriented (*start*) over avoidance-oriented (*stop*) framing, affirmative (*support*) over oppositional (*oppose*) framing, and *increase* over *decrease* framing of semantically equivalent statements (Figure 4). The strongest consistent preferences across ideology groups were for *support* and *increase* framings: preference for *support* framing ranged from 74.0% (95% CI, 65.7%-82.4%) for very liberal respondents to 79.5% (95% CI, 73.3%-85.7%) for liberal respondents (*P* = .008), and preference for *increase* framing ranged from 72.7% (95% CI, 64.0%-81.5%) for very conservative respondents) to 82.1% (95% CI, 76.2%-88.0%) for liberal respondents (*P* = .33). These framings showed high endorsement across ideology groups with narrow ranges of preference compared with the other 2 framing contrasts.

Political ideology differentiated strength of preference for collectivist and action-oriented framing. Preference for the *we* framing was highest among very liberal (83.3% [95% CI, 76.2%-90.4%]) and liberal respondents (81.4% [95% CI, 75.5%-87.4%]) compared with conservative (60.2% [95% CI, 53.5%-66.9%]) and very conservative respondents (47.3% [95% CI, 37.4%-57.1%]) ( *P* < .001). Similarly, preference for *start* framing differed by ideology, with stronger endorsement among liberal (83.1% [95% CI, 77.3%-88.8%]) and very liberal respondents (83.5% [95% CI, 76.4%-90.6%]) than among conservative (66.4% [95% CI, 60.0%-72.9%]) and very conservative respondents (65.8% [95% CI, 56.4%-75.1%]) (*P* < .001).

## Discussion

Our study supports previous reports that have shown there are strong differences in language and framing preferences across political ideological groups when it comes to health communication.^10,26,27^ Using embedded randomized experiments, we specifically sought to test acceptability of definitions and their alignment with personal and perceived American values, evaluative reactions to common terms used in health equity work, and framing preference for public health messages in a three-module survey.

In module 1, respondents across ideological groups judged both *health equity* and *health equality* as consistent with their personal values. Values alignment was higher for the *health equity* definition than for the *health equality* definition. Perceived alignment between definitions and core American values varied across political ideology. Taken together, these findings suggest that despite differing political perspectives, there is a shared recognition of fairness and opportunity in health as guiding principles and that equity language resonates personally, even when audiences are uncertain that it represents a shared civic norm. An important finding was the divergence between personal beliefs and the respondent’s beliefs about core American values. Liberal and very liberal respondents demonstrated a sharp distinction between personal and core American values, while very conservative respondents reported closer alignment between personal and core American values. Even among conservative and very conservative groups, there was lower endorsement of either definition as a core American value, suggesting that across ideological groups, individuals perceive a gap between personal beliefs and perceptions of national norms. Prior work in political psychology has shown that misperceptions about where the broader public stands can amplify perceived polarization and increase the sense that policy issues are more ideologically divisive than they truly are.^28^ Recognizing and addressing this perceived misalignment may be a critical component of effective public health communication. Taken together, these results may help identify opportunities for bridging conversations through values-based language, presented clearly and with context, to facilitate resonance across ideological lines. For health communication across ideological identities, a pragmatic approach may be to lead with widely shared values, then subsequently link those values to concrete actions (ie, health care coverage, timely care, prevention) without partisan cues. These findings are supported by previous work showing that values-based policy framing increases support across political ideologies without backlash from other groups.^29,30^

In module 2, we looked at reactions toward commonly used terms in health equity discourse. We found that several terms elicited overwhelmingly positive responses (*accessible health care* and *health care investment*). Even when statistically significant ideological differences emerged—specifically, more negative reactions from very conservative respondents to *population health* and *community health—*more than 85% still viewed these terms positively or neutrally, suggesting that these terms remain largely unifying rather than polarizing. A second cluster of terms elicited more varied evaluative reactions across political ideologies, reflecting language that may be perceived as less neutral or more ideologically charged. For example, *inclusive health care* showed a clear ideological gradient, with only 4.3% of very liberal respondents reacting negatively compared with 32.7% of very conservative respondents. While most respondents across all groups still rated the term as neutral or positive, this divergence suggests that phrases invoking inclusion may carry political connotations for some audiences. Similarly, very conservative and conservative respondents had more negative reactions to the term *marginalized communities*. In contrast, liberal respondents provided unexpectedly greater negative ratings for terms such as *health disparities* and *differences in health outcomes*. This may reflect fatigue or skepticism in driving structural change or a reaction to the underlying issues themselves rather than the terminology. Although no terms were wholly rejected, some terms may not have the same resonance across people with differing political ideologies. In ideologically diverse settings, terms that elicit more variable reactions may benefit from contextual framing or coupling with shared values to reduce ambiguity and potential resistance. The terms that elicited consistently nonnegative reactions across ideology groups map onto policy domains that have historically attracted bipartisan support. Although our study did not evaluate policy preferences directly, prior evidence shows that population health initiatives such as Medicaid expansion have gained broad public legitimacy across states and party lines, and investments in core public health infrastructure receive support across political contexts.^31^ US global health programs—including US Agency for International Development and the President’s Emergency Plan for AIDS Relief—are further examples of health-directed investments that have maintained bipartisan backing over time.^32,33^ These domains may represent areas of shared interest where health system strengthening and community well-being align with public values across the public spectrum.

In module 3 of our study, we examined how subtle shifts in framing of public health messages affect respondent preferences across political ideologies. Although ideological gradients were observed, respondents across all groups largely favored collectivist language over individual-focused phrasing. Although community-oriented appeals are often perceived as less resonant with conservative audiences, our findings indicate that reactions were not uniform and showed meaningful variation within ideology groups.^34^

For the remaining paired statements, most respondents across all ideological groups preferred approach-oriented, positive framing. At least 65% of respondents in each ideological group favored statements using *support*, *increase*, and *start* framing over statements using *oppose*, *decrease*, and *stop* framing. These results reinforce evidence that affirmatively framed, approach-oriented messages elicit broader appeal than negative- or avoidance-based alternatives and highlight a pathway for building consensus around health equity communication across diverse audiences.^35,36,37^

### Limitations

Our results must be interpreted considering several limitations. Although the YouGov methodology has been extensively validated and shown to produce samples that closely match US population benchmarks, recruitment from a nonprobability-based online panel may be subject to selection and coverage biases (eg, differential internet access), and the findings are to be interpreted as descriptive, particular for subgroup estimates. Despite matching and weighting to national benchmarks, respondents may differ from the broader US population in unmeasured ways. Self-reported ideology and evaluative reactions may also be imprecise, as respondents interpret ideological labels differently and may vary in how they react to terminology without additional context. Second, the surprising negative responses among liberal and very liberal respondents to certain health equity terms are difficult to interpret, as we did not distinguish whether these reflected disapproval of the terminology, frustration with the issues, or another source of negative reactions, nor did we test comprehension to ensure participants fully understood the contrasts. Finally, as with all survey research, expressed preferences may not translate to actual behaviors, where context, messenger identity, and repeated exposure shape communication effects.

Although this study focused on how language and framing shape receptiveness to health equity concepts in the wake of policy changes, it is important to acknowledge that health equity has long been embedded in political discourse. Advocates for equity have historically engaged in social and political movements aimed at addressing structural determinants of health, and public health institutions themselves are not ideologically neutral actors. Communication strategies alone cannot resolve deeper disagreements about the substantive goals of health equity, nor should they be substitutes for genuine engagement with communities whose values and policy preferences may differ from those held by many in the public health community. Recognizing these long-standing political dimensions provides necessary context for interpreting our findings and underscores that effective communication must be paired with responsiveness to diverse perspectives in a pluralistic society.

## Conclusions

In this cross-sectional online survey study of 1000 US adults, ideological differences were observed in reactions to specific health equity terms, yet several commonly used terms and affirmative, collective framings were viewed favorably across groups. These findings may help public health professionals and advocates shape language that connects more deeply and inclusively with broad audiences, regardless of political orientation.^38^ As debates over health and equity increasingly intersect with political identity, these data provide actionable insights for clinicians, researchers, policymakers, and advocates. Strategic language and framing anchored in shared values and constructive framing offer a pathway to advance public health communication that is both principled and broadly resonant. Future efforts should embed more intentional language in public health messaging and policy debates, with testing and refinement in real-world contexts to build trust, engage diverse constituencies, and advance equity in health.

## Notes

Supplement 1. **eTable.** Comparison of Demographic Distribution of YouGov Sample Compared With Data From the American Community Survey (ACS) Conducted by the US Census Bureau **eAppendix.** Study Survey

Supplement 2.
Data Sharing Statement

## References

[1] Public health infrastructure in crisis: HHS workforce cuts, reorganizations, and funding reductions: impacts and solutions. Trust for America’s Health. Accessed December 18, 2025. https://www.tfah.org/report-details/funding-report-2025/

[2] Greer SL, Jarman H, Kulikoff R, Yaver M. Trump’s second presidency begins: evaluating effects on the US health system. Lancet Reg Health Am. 2025;48:101173. doi:10.1016/j.lana.2025.101173 40678372 PMC12270643

[3] Wang SX, Eniasivam A, Sterken D, ; the HOMERUN HEARS Work Group and the HOMERUN Collaborative. Navigating uncertainty: sustaining health equity in a shifting landscape. J Gen Intern Med. Published online November 17, 2025. doi:10.1007/s11606-025-09874-z 41247421 PMC13009315

[4] Eick SM, Eatman JA, Chandler M, Brooks NR. Reproductive and social policies, sociopolitical stress, and implications for maternal and child health equity. Curr Environ Health Rep. 2024;11(2):279-287. doi:10.1007/s40572-024-00443-w 38639910 PMC11531301

[5] Leider JP, Resnick B, Bishai D, Scutchfield FD. How much do we spend? creating historical estimates of public health expenditures in the United States at the federal, state, and local levels. Annu Rev Public Health. 2018;39:471-487. doi:10.1146/annurev-publhealth-040617-013455 29346058

[6] Frieden TR. Dismantling public health infrastructure, endangering American lives. N Engl J Med. 2025;393(7):625-627. doi:10.1056/NEJMp2509087 40737612

[7] Oberlander J. Progress lost—the unraveling of Medicaid and the Affordable Care Act. N Engl J Med. 2025;393(7):628-629. doi:10.1056/NEJMp2509768 40737607

[8] Duggan CP, Bhutta ZA. “Putting America First”—undermining health for populations at home and abroad. N Engl J Med. 2025;392(18):1769-1771. doi:10.1056/NEJMp2503243 40239090

[9] Cené CW. The health equity, medical, and scientific costs of dismantling DEI. N Engl J Med. 2025;392(24):2396-2399. doi:10.1056/NEJMp2506286 40544437

[10] Rodriguez HP, Laugesen MJ, Watts CA. A randomized experiment of issue framing and voter support of tax increases for health insurance expansion. Health Policy. 2010;98(2-3):245-255. doi:10.1016/j.healthpol.2010.06.020 20655125

[11] Chong D, Druckman JN. Framing theory. Annu Rev Polit Sci. 2007;10(1):103-126. doi:10.1146/annurev.polisci.10.072805.103054

[12] Gallagher KM, Updegraff JA. Health message framing effects on attitudes, intentions, and behavior: a meta-analytic review. Ann Behav Med. 2012;43(1):101-116. doi:10.1007/s12160-011-9308-7 21993844

[13] Dixon G, Hmielowski J, Ma Y. Improving climate change acceptance among US conservatives through value-based message targeting. Sci Commun. 2017;39(4):520-534. doi:10.1177/1075547017715473

[14] Gollust SE, Cappella JN. Understanding public resistance to messages about health disparities. J Health Commun. 2014;19(4):493-510. doi:10.1080/10810730.2013.821561 24417451

[15] Voelkel JG, Willer R. Resolving the progressive paradox: conservative value framing of progressive economic policies increases candidate support. *SSRN*. Published online May 13, 2019. doi:10.2139/ssrn.3385818

[16] Weiss M, Zoorob M. Political frames of public health crises: discussing the opioid epidemic in the US Congress. Soc Sci Med. 2021;281:114087. doi:10.1016/j.socscimed.2021.114087 34102424

[17] Del Pozo B, Rouhani S, Bailey A, . The effects of message framing on US police chiefs’ support for interventions for opioid use disorder: a randomized survey experiment. Health Justice. 2024;12(1):50. doi:10.1186/s40352-024-00306-4 39699824 PMC11660544

[18] Gollust SE, Niederdeppe J, Barry CL. Framing the consequences of childhood obesity to increase public support for obesity prevention policy. Am J Public Health. 2013;103(11):e96-e102. doi:10.2105/AJPH.2013.301271 24028237 PMC3828688

[19] Cullerton K, Demeshko A, Waller M. Effect of message framing on support for a sugar-sweetened beverage tax in Australia: a cross-sectional survey analysis. Health Promot Int. 2024;39(1):daad193. doi:10.1093/heapro/daad193 38206788 PMC10783238

[20] Roh S, Niederdeppe J. The word outside and the pictures in our heads: contingent framing effects of labels on health policy preferences by political ideology. Health Commun. 2016;31(9):1063-1071. doi:10.1080/10410236.2015.1037420 26799756

[21] Doell KC, Pärnamets P, Harris EA, Hackel LM, Van Bavel JJ. Understanding the effects of partisan identity on climate change. Curr Opin Behav Sci. 2021;42:54-59. doi:10.1016/j.cobeha.2021.03.013

[22] Kaplan JT, Vaccaro A, Henning M, Christov-Moore L. Moral reframing of messages about mask-wearing during the COVID-19 pandemic. Sci Rep. 2023;13(1):10140. doi:10.1038/s41598-023-37075-3 37349385 PMC10287646

[23] Callegaro M, DiSogra C. Computing response metrics for online panels. Public Opin Q. 2008;72(5):1008-1032. doi:10.1093/poq/nfn065

[24] Methods overview. Pew Research Center. 2023. Accessed December 18, 2025. https://www.pewresearch.org/our-methods/

[25] American Community Survey research and methodology. US Census Bureau. 2023. Accessed January 29, 2026. https://www.census.gov/programs-surveys/acs/methodology.html

[26] Rothman AJ, Salovey P. Shaping perceptions to motivate healthy behavior: the role of message framing. Psychol Bull. 1997;121(1):3-19. doi:10.1037/0033-2909.121.1.3 9000890

[27] Beall JM, Casola WR, Peterson MN, . Cultural cognition and ideological framing influence communication about Zoonotic disease in the era of COVID-19. Front Commun (Lausanne). 2021;6:645692. doi:10.3389/fcomm.2021.645692

[28] Ahler DJ, Sood G. The parties in our heads: misperceptions about party composition and their consequences. J Polit. 2018;80(3):964-981. doi:10.1086/697253

[29] Voelkel JG, Mernyk JS, Willer R. Moral reframing increases support for economically progressive candidates. PSNA Nexus. 2023;2(6):pgad154. doi:10.1093/pnasnexus/pgad154 PMC10281394 37346269

[30] Voelkel JG, Stagnaro MN, Chu JY, . Megastudy testing 25 treatments to reduce antidemocratic attitudes and partisan animosity. Science. 2024;386(6719):eadh4764. doi:10.1126/science.adh4764 39418366

[31] DeSilver D. What the data says about Medicaid. Pew Research Center. June 24, 2025. Accessed December 18, 2025. https://www.pewresearch.org/short-reads/2025/06/24/what-the-data-says-about-medicaid/

[32] Noon MJ, She X, Meier BM. The US elections as a determinant of global health. Lancet Reg Health Am. 2024;39:100884. doi:10.1016/j.lana.2024.100884 39309538 PMC11416580

[33] Fauci AS, Eisinger RW. PEPFAR—15 years and counting the lives saved. N Engl J Med. 2018;378(4):314-316. doi:10.1056/NEJMp1714773 29365298

[34] Kidwell B, Farmer A, Hardesty DM. Getting liberals and conservatives to go green: political ideology and congruent appeals. J Consum Res. 2013;40(2):350-367. doi:10.1086/670610

[35] Catapano R, Tormala ZL. Do I support that it’s good or oppose that it’s bad? the effect of support-oppose framing on attitude sharing. J Pers Soc Psychol. 2021;121(1):23-42. doi:10.1037/pspa0000266 33617285

[36] Hussein MA, Tormala ZL. You versus we: how pronoun use shapes perceptions of receptiveness. J Exp Soc Psychol. 2024;110:104555. doi:10.1016/j.jesp.2023.104555

[37] Lee C, Bechler CJ, Tormala ZL. Increase versus decrease framing: how framing a finding as an increase boosts its perceived magnitude. J Exp Psychol Gen. 2025;154(10):2874-2894. doi:10.1037/xge0001818 40788698

[38] Haidt J. The Righteous Mind: Why Good People Are Divided by Politics and Religion. Pantheon Books; 2012.

