# Supplementary material for: Preferences for Shared Language for Health Equity Across the Political Spectrum
Source: JAMA Netw Open. 2026 Mar 6;9(3):e260277. doi: 10.1001/jamanetworkopen.2026.0277 (PMC12966926; doi:10.1001/jamanetworkopen.2026.0277)
Supplement: Supplement 1. — eTable. Comparison of Demographic Distribution of YouGov Sample Compared With Data From the American Community Survey (ACS) Conducted by the US Census Bureau eAppendix. Study Survey [file jamanetwopen-e260277-s001.pdf]

Supplementary Online Content

Wang SXY, Song S, Nikolov MC, Tormala Z, Kaplan RM, Schulman K. Preferences for shared language for health equity across the political spectrum. *JAMA Netw Open.* 2026;9(3):e260277. doi:10.1001/jamanetworkopen.2026.0277

eTable. Comparison of Demographic Distribution of YouGov Sample Compared With Data From the American Community Survey (ACS) Conducted by the US Census Bureau

eAppendix. Study Survey

This supplementary material has been provided by the authors to give readers additional information about their work.

© 2026 Wang SXY et al. *JAMA Network Open*.

eTable. Comparison of Demographic Distribution of YouGov Sample Compared With Data From the American Community Survey (ACS) Conducted by the US Census Bureau

Columns within categories may not sum to 100% because of rounding errors.

Attribute	Question	Level	weighted YouGov %	ACS %
Age Range	In what year were you born?	18-24	11%	12%
		25-34	17%	18%
		35-44	18%	18%
		45-54	14%	16%
		55-64	18%	17%
		65-74	15%	14%
		75+	7%	6%
Gender	Are you...?	Male	49%	49%
		Female	51%	51%
Race/Ethnicity	What racial or ethnic group best describes you?	Asian or Asian-American	3%	62%
		Black or African-American	12%	12%
		Hispanic or Latino	11%	16%
		Middle Eastern	0.4%	7%
		Native American	2%	1%
		White	66%	0%
		Two or more races	3%	3%
		Other (unspecified)	3%	1%
Marital Status	What is your marital status?	Married/Partnered	53%	54%
		Previously married	18%	23%
		Never married	30%	23%
Education	What is the highest level of education you have completed?	Did not graduate from HS	7%	10%
		High school graduate	31%	27%
		Some college/Associates degree	28%	28%
		Bachelors degree	21%	23%
		Postgraduate degree (MA,MBA,MD,JD,PhD,etc.)	12%	12%

© 2026 Wang SXY et al. *JAMA Network Open*.

eAppendix. Study Survey

Page: implicit_page_sample_type

SINGLE CHOICE
Sample type

varlabel Sample Type

1 ○ national respondents
2 ○ conservative OS
8 *Skipped*
9 *Not Asked*

© 2026 Wang SXY et al. *JAMA Network Open*.

Page: testing_pg

SINGLE CHOICE
For testing purposes only, please select at treatment condition for Q3 (participants will be randomly assigned)

varlabel STAN0189 Q3 wording treatment condition

1 ○ Health equity
2 ○ Health equality
8 *Skipped*
9 *Not Asked*

SINGLE CHOICE
For testing purposes only, please select at statement for Topic 1 (participants will be randomly assigned)

varlabel STAN0189 health topic treatment condition 1

1 ○ Statement 1
2 ○ Statement 2
8 *Skipped*
9 *Not Asked*

SINGLE CHOICE
For testing purposes only, please select at statement for Topic 2 (participants will be randomly assigned)

varlabel STAN0189 health topic treatment condition 2

1 ○ Statement 1
2 ○ Statement 2
8 *Skipped*
9 *Not Asked*

SINGLE CHOICE
For testing purposes only, please select at statement for Topic 3 (participants will be randomly assigned)

varlabel STAN0189 health topic treatment condition 3

1 ○ Statement 1
2 ○ Statement 2
8 *Skipped*
9 *Not Asked*

© 2026 Wang SXY et al. *JAMA Network Open*.

SINGLE CHOICE
For testing purposes only, please select at statement for Topic 4 (participants will be randomly assigned)

varlabel STAN0189 health topic treatment condition 4

1 ○ Statement 1
2 ○ Statement 2
8 *Skipped*
9 *Not Asked*

SINGLE CHOICE
For testing purposes only, please select at statement for Topic 5 (participants will be randomly assigned)

varlabel STAN0189 health topic treatment condition 5

1 ○ Statement 1
2 ○ Statement 2
8 *Skipped*
9 *Not Asked*

© 2026 Wang SXY et al. *JAMA Network Open*.

Page: implicit_page_ideo5

SINGLE CHOICE
In general, how would you describe your own political viewpoint?

varlabel Ideology

1 ○ Very liberal
2 ○ Liberal
3 ○ Moderate
4 ○ Conservative
5 ○ Very conservative
6 ○ Not sure *Not randomized*
8 *Skipped*
9 *Not Asked*

© 2026 Wang SXY et al. *JAMA Network Open*.

Page: consent_pg

<p>DESCRIPTION: You are invited to participate in a research study on preferred language and terms to describe health equity concepts. You will be asked to complete a survey that asks for your preferences across different terms and ways of describing health equity and how it aligns with your personal values. Participation in this research is voluntary, and you are free to withdraw your consent at any time. </p> <p>TIME INVOLVEMENT: Your participation will take approximately 10-15 minutes. </p> <p>PAYMENTS: You will receive no direct payment for your participation. </p> <p>PRIVACY AND CONFIDENTIALITY: The risks associated with this study are minimal. Study data will be stored securely, in compliance with Stanford University standards, minimizing the risk of confidentiality breach. Your individual privacy will be maintained during the research and in all published and written data resulting from the study. </p> <p>CONTACT INFORMATION:</p> <p><i>Questions:</i> If you have any questions, concerns or complaints about this research, its procedures, risks and benefits, contact the Protocol Director. </p> <p>Independent Contact: If you are not satisfied with how this study is being conducted, or if you have any concerns, complaints, or general questions about the research or your rights as a participant, please contact the Stanford Institutional Review Board (IRB) to speak to someone independent of the research team at 650-723-5244 or toll free at 1-866-680-2906. You can also write to the Stanford IRB, Stanford University, 1705 El Camino Real, Palo Alto, CA 94306.</p> <p>Please save or print a copy of this page for your records. </p>

SINGLE CHOICE
If you agree to participate in this research, please indicate by selecting from the choices below (Yes for agree, No for disagree).

varlabel Consent

1 ○ Yes
2 ○ No
8 *Skipped*
9 *Not Asked*

© 2026 Wang SXY et al. *JAMA Network Open*.

Page: values_pg

This survey is interested in understanding your response to different terms around health care. To begin, we will define the term health equity/health equality.

Health equity means ensuring that everyone has the opportunity to achieve their best possible health by addressing differences in access to care, resources, and support. It recognizes that people have different circumstances, and some may need more assistance than others to reach the same health outcomes.

Health equality means providing the same resources to everyone, regardless of their individual needs or circumstances. This approach ensures uniform distribution, but may not always lead to equal health outcomes if people start from different places or face different challenges.

GRID
In the matrix below, you will see several different statements on the left. Please rate how the statements match up with your own values.

varlabel Personal values on health terms

ROWS

Q3_a- *prompt once on skip*	**Healthy equity/Health equality** aligns with my personal values
Q3_b- *prompt once on skip*	Discussions about **healthy equity/health equality** make me feel frustrated
Q3_c- *prompt once on skip*	**Healthy equity/Health equality** reflects a core American value
Q3_d- *prompt once on skip*	**Healthy equity/Health equality** seems unfair or misguided

COLUMNS

1 ○ Strongly Disagree
2 ○ Disagree
3 ○ Neutral
4 ○ Agree
5 ○ Strongly Agree
8 *Skipped*
9 *Not Asked*

© 2026 Wang SXY et al. *JAMA Network Open*.

Page: health_preference_intro_pg

<p><i>The next exercise asks about your reactions to several statements about health care. First, read the statement. Below each statement, you will be asked a series of questions on your reaction to the statements. </i></p>

© 2026 Wang SXY et al. *JAMA Network Open*.

Page: health_topic1_pg

Statement 1: Everyone should have a fair chance to live a healthy life. This means ensuring that access to care is based on equal opportunity, without special treatment for certain groups.

Statement 2: Everyone should have a fair chance to live a healthy life. This means actively removing barriers that prevent many from accessing the care they need, ensuring real fairness for all.

SINGLE CHOICE
How strongly do you agree?

varlabel Health reframed as fairness in opportunity vs. health equity - Agreement

1 ○ Strongly Disagree
2 ○ Disagree
3 ○ Neutral
4 ○ Agree
5 ○ Strongly Agree
8 *Skipped*
9 *Not Asked*

SINGLE CHOICE
How well does this statement align with your personal values?

varlabel Health reframed as fairness in opportunity vs. health equity - Personal Values

1 ○ Does not align at all
2 ○ Mostly does not align
3 ○ Neutral
4 ○ Mostly aligns
5 ○ Completely aligns
8 *Skipped*
9 *Not Asked*

SINGLE CHOICE
Now, think about Americans as a whole and their values as a society. How does this statement align with those values?

varlabel Health reframed as fairness in opportunity vs. health equity - American Values

1 ○ Does not align at all
2 ○ Mostly does not align
3 ○ Neutral
4 ○ Mostly aligns
5 ○ Completely aligns
8 *Skipped*
9 *Not Asked*

© 2026 Wang SXY et al. *JAMA Network Open*.

Page: health_topic2_pg

Statement 1: A healthy population is the backbone of a strong economy. This means promoting personal accountability and market-driven solutions to reduce waste, lower costs, and boost productivity.

Statement 2: A healthy population is the backbone of a strong economy. This means investing in public health and preventive services to reduce inequality and create shared prosperity for everyone.

SINGLE CHOICE
How strongly do you agree?

varlabel Health Equity as Economic Stability and Efficiency - Agreement

1 ○ Strongly Disagree
2 ○ Disagree
3 ○ Neutral
4 ○ Agree
5 ○ Strongly Agree
8 *Skipped*
9 *Not Asked*

SINGLE CHOICE
How well does this statement align with your personal values?

varlabel Health Equity as Economic Stability and Efficiency - Personal Values

1 ○ Does not align at all
2 ○ Mostly does not align
3 ○ Neutral
4 ○ Mostly aligns
5 ○ Completely aligns
8 *Skipped*
9 *Not Asked*

SINGLE CHOICE
Now, think about Americans as a whole and their values as a society. How does this statement align with those values?

varlabel Health Equity as Economic Stability and Efficiency - American Values

1 ○ Does not align at all
2 ○ Mostly does not align
3 ○ Neutral
4 ○ Mostly aligns
5 ○ Completely aligns
8 *Skipped*
9 *Not Asked*

© 2026 Wang SXY et al. *JAMA Network Open*.

Page: health_topic3_pg

Statement 1: A strong America means strong and healthy individuals. Ensuring access to healthcare helps keep our nation competitive and resilient.

Statement 2: A strong America means strong and healthy individuals. That means addressing longstanding barriers to care and providing resources where they're needed most, ensuring every community can thrive and our nation's strength is shared by all.

SINGLE CHOICE
How strongly do you agree?

varlabel Appealing to Patriotism and National Strength - Agreement

1 ○ Strongly Disagree
2 ○ Disagree
3 ○ Neutral
4 ○ Agree
5 ○ Strongly Agree
8 *Skipped*
9 *Not Asked*

SINGLE CHOICE
How well does this statement align with your personal values?

varlabel Appealing to Patriotism and National Strength - Personal Values

1 ○ Does not align at all
2 ○ Mostly does not align
3 ○ Neutral
4 ○ Mostly aligns
5 ○ Completely aligns
8 *Skipped*
9 *Not Asked*

© 2026 Wang SXY et al. *JAMA Network Open*.

SINGLE CHOICE

Now, think about Americans as a whole and their values as a society. How does this statement align with those values?

varlabel Appealing to Patriotism and National Strength - American Values

1 ○ Does not align at all
2 ○ Mostly does not align
3 ○ Neutral
4 ○ Mostly aligns
5 ○ Completely aligns
8 *Skipped*
9 *Not Asked*

© 2026 Wang SXY et al. *JAMA Network Open*.

Page: health_topic4_pg

Statement 1. Every person's health matters. Personal choices and actions are the foundation of good health.

Statement 2: Every person's health matters. We have a shared responsibility to help individuals and families achieve good health.

SINGLE CHOICE
How strongly do you agree?

varlabel Individual vs. Collective Responsibility - Agreement

1 ○ Strongly Disagree
2 ○ Disagree
3 ○ Neutral
4 ○ Agree
5 ○ Strongly Agree
8 *Skipped*
9 *Not Asked*

SINGLE CHOICE
How well does this statement align with your personal values?

varlabel Individual vs. Collective Responsibility - Personal Values

1 ○ Does not align at all
2 ○ Mostly does not align
3 ○ Neutral
4 ○ Mostly aligns
5 ○ Completely aligns
8 *Skipped*
9 *Not Asked*

SINGLE CHOICE
Now, think about Americans as a whole and their values as a society. How does this statement align with those values?

varlabel Individual vs. Collective Responsibility - American Values

1 ○ Does not align at all
2 ○ Mostly does not align
3 ○ Neutral
4 ○ Mostly aligns
5 ○ Completely aligns
8 *Skipped*
9 *Not Asked*

© 2026 Wang SXY et al. *JAMA Network Open*.

Page: health_topic5_pg

Statement 1: Healthy communities benefit everyone. To do this work, we should use non-partisan, neutral language that emphasizes solutions and focuses on unifying goals without labels that may polarize.

Statement 2: Healthy communities benefit everyone. This means speaking openly about the systemic forces behind inequities, using direct language to name and challenge structural injustices—even if that language disrupts comfortable narratives.

SINGLE CHOICE
How strongly do you agree?

varlabel Avoiding Divisive Terminology - Agreement

1 ○ Strongly Disagree
2 ○ Disagree
3 ○ Neutral
4 ○ Agree
5 ○ Strongly Agree
8 *Skipped*
9 *Not Asked*

SINGLE CHOICE
How well does this statement align with your personal values?

varlabel Avoiding Divisive Terminology - Personal Values

1 ○ Does not align at all
2 ○ Mostly does not align
3 ○ Neutral
4 ○ Mostly aligns
5 ○ Completely aligns
8 *Skipped*
9 *Not Asked*

SINGLE CHOICE
Now, think about Americans as a whole and their values as a society. How does this statement align with those values?

varlabel Avoiding Divisive Terminology - American Values

1 ○ Does not align at all
2 ○ Mostly does not align
3 ○ Neutral
4 ○ Mostly aligns
5 ○ Completely aligns
8 *Skipped*
9 *Not Asked*

© 2026 Wang SXY et al. *JAMA Network Open*.

Page: healthcare_topic6_pg

<p>Below are two statements about improving healthcare access. Please indicate which statement resonates more with you or if you have no preference.</p>

SINGLE CHOICE
Statement 1: **Your** health matters. **You** deserve access to good healthcare. ***Statement 2:*** **Our** health matters. **We all** deserve access to good healthcare.

varlabel You vs. We

1 ○ Statement 1
2 ○ Statement 2
3 ○ No Preference
8 *Skipped*
9 *Not Asked*

© 2026 Wang SXY et al. *JAMA Network Open*.

Page: healthcare_topic7_pg

<p>Below are two statements about improving healthcare access. Please indicate which statement resonates more with you or if you have no preference.</p>

SINGLE CHOICE
Statement 1: We need to **support** efforts to **improve** healthcare access and quality. ***Statement 2:*** We need to **oppose** efforts to **limit** healthcare access and quality.

varlabel Supportive vs. Oppositional Framing

1 ○ Statement 1
2 ○ Statement 2
3 ○ No Preference
8 *Skipped*
9 *Not Asked*

© 2026 Wang SXY et al. *JAMA Network Open*.

Page: healthcare_topic8_pg

<p>Below are two statements about improving healthcare access. Please indicate which statement resonates more with you or if you have no preference.</p>

SINGLE CHOICE
Statement 1: **Increasing** affordability and access to healthcare will **boost** productivity and **strengthen** the economy. ***Statement 2:*** **Decreasing** affordability and access to healthcare will **reduce** productivity and **weaken** the economy.

varlabel Increase vs. Decrease Framing

1 ○ Statement 1
2 ○ Statement 2
3 ○ No Preference
8 *Skipped*
9 *Not Asked*

© 2026 Wang SXY et al. *JAMA Network Open*.

Page: healthcare_topic9_pg

<p>Below are two statements about improving healthcare access. Please indicate which statement resonates more with you or if you have no preference.</p>

SINGLE CHOICE
Statement 1: Improving healthcare access for all will help people **start** living **healthier** lives.
Statement 2: Improving healthcare access for all will help people **stop** living **un**healthy lives.

varlabel Stop vs. Start

1 ○ Statement 1
2 ○ Statement 2
3 ○ No Preference
8 *Skipped*
9 *Not Asked*

© 2026 Wang SXY et al. *JAMA Network Open*.

Page: implicit_page_preferred_terms

DYNAMIC GRID
You will see two terms related to health. For each term, select which makes health care concerns seem more relevant to you:

varlabel Select preferred terms

ROWS

preferred_terms_a- *prompt once on skip*	A) Healthcare is a right vs. B) Healthcare is a privilege
preferred_terms_b- *prompt once on skip*	<p>A) Health care is personal responsibility<p><p>vs.</p><p>B) Health care is a collective responsibility</p>
preferred_terms_c- *prompt once on skip*	A) Equality of health opportunity vs. B) Equality of health outcomes
preferred_terms_d- *prompt once on skip*	A) Marginalized communities vs. B) Vulnerable populations
preferred_terms_e- *prompt once on skip*	A) Health disparities vs. B) Differences in health outcomes
preferred_terms_f- *prompt once on skip*	A) Barriers to care vs. B) Systemic inequality
preferred_terms_g- *prompt once on skip*	A) Accessible health care vs. B) Removing barriers to care

COLUMNS

1 ○ Statement A
2 ○ Statement B
3 ○ Neither
4 ○ Both
8 *Skipped*
9 *Not Asked*

© 2026 Wang SXY et al. *JAMA Network Open*.

Page: implicit_page_preferred_terms_response_scale_a

SCALE
Select whether these terms elicit a positive, negative, or neutral response from you.
Barriers to care

min	-3
varlabel	Response to preferred terms scale - Barriers to care
max	3

© 2026 Wang SXY et al. *JAMA Network Open*.

Page:implicit_page_preferred_terms_response_scale_b

SCALE
Select whether these terms elicit a positive, negative, or neutral response from you.
Marginalized communities

min	-3
varlabel	Response to preferred terms scale - Marginalized communities
max	3

© 2026 Wang SXY et al. *JAMA Network Open*.

Page: implicit_page_preferred_terms_response_scale_c

SCALE
Select whether these terms elicit a positive, negative, or neutral response from you.
Vulnerable populations

min	-3
varlabel	Response to preferred terms scale - Vulnerable populations
max	3

© 2026 Wang SXY et al. *JAMA Network Open*.

Page: implicit_page_preferred_terms_response_scale_d

SCALE
Select whether these terms elicit a positive, negative, or neutral response from you.
Health disparities

min	-3
varlabel	Response to preferred terms scale - Health disparities
max	3

© 2026 Wang SXY et al. *JAMA Network Open*.

Page: implicit_page_preferred_terms_response_scale_e

SCALE
Select whether these terms elicit a positive, negative, or neutral response from you.
Differences in health outcomes

min	-3
varlabel	Response to preferred terms scale - Differences in health outcomes
max	3

© 2026 Wang SXY et al. *JAMA Network Open*.

Page: implicit_page_preferred_terms_response_scale_f

SCALE
Select whether these terms elicit a positive, negative, or neutral response from you.
Accessible health care

min	-3
varlabel	Response to preferred terms scale - Accessible health care
max	3

© 2026 Wang SXY et al. *JAMA Network Open*.

Page: implicit_page_preferred_terms_response_scale_g

SCALE
Select whether these terms elicit a positive, negative, or neutral response from you.
Health care investment

min	-3
varlabel	Response to preferred terms scale - Health care investment
max	3

© 2026 Wang SXY et al. *JAMA Network Open*.

Page: implicit_page_preferred_terms_response_scale_h

SCALE
Select whether these terms elicit a positive, negative, or neutral response from you.
Inclusive health care

min	-3
varlabel	Response to preferred terms scale - Inclusive health care
max	3

© 2026 Wang SXY et al. *JAMA Network Open*.

Page: implicit_page_preferred_terms_response_scale_i

SCALE
Select whether these terms elicit a positive, negative, or neutral response from you.
Population health

min	-3
varlabel	Response to preferred terms scale - Population health
max	3

© 2026 Wang SXY et al. *JAMA Network Open*.

Page: implicit_page_preferred_terms_response_scale_j

SCALE
Select whether these terms elicit a positive, negative, or neutral response from you.
Community health

min	-3
varlabel	Response to preferred terms scale - Community health
max	3

© 2026 Wang SXY et al. *JAMA Network Open*.

Page: implicit_page_terms

OPEN TEXTBOX
We are exploring ways to describe programs and initiatives that aim to improve health across communities and in the country. Are there other words or phrases to **describe health** that you would suggest **using in communications**?

dk_text	I don't have other terms to suggest
varlabel	Suggest health words and phrases

© 2026 Wang SXY et al. *JAMA Network Open*.

Page: implicit_page_health_events

MULTIPLE CHOICE
In the past 12 months, have you experienced any of the following serious health events?

varlabel Serious health events

1 ☐ Hospitalization overnight or longer
2 ☐ Major surgery requiring prolonged recovery time
3 ☐ Emergency medical care for a life threatening condition
4 ☐ Diagnosis of a serious health condition (eg. cancer, heart attack, stroke)
5 ☐ None of the above *Not randomized,exclude other punches*

© 2026 Wang SXY et al. *JAMA Network Open*.

Page: implicit_page_genhealth

SINGLE CHOICE
Would you say your health in general is excellent, very good, good, fair, or poor?

varlabel General health condition

1 ○ Excellent
2 ○ Very good
3 ○ Good
4 ○ Fair
5 ○ Poor
8 *Skipped*
9 *Not Asked*

© 2026 Wang SXY et al. *JAMA Network Open*.

Page: implicit_page_healthinsur_srg

MULTIPLE CHOICE
Are you currently covered by any of the following types of health insurance or health coverage plans?

varlabel Types of health insurance or coverage plans

1 ☐ Insurance through a current or former employer or union, or insurance through a spouse's/ other family member's employer
2 ☐ Insurance purchased directly from an insurance company
3 ☐ Medicare, for people 65 and older, or people with certain disabilities
4 ☐ Medicaid, Medical Assistance, or any kind of government-assistance plan for those with low incomes or a disability
5 ☐ TRICARE or other military health care
6 ☐ VA (including those who have ever used or enrolled for VA health care)
7 ☐ Indian Health Service
8 ☐ Any other type of health insurance or health coverage plan
99 ☐ Not applicable, not covered by health insurance currently — *Exclude other punches*

© 2026 Wang SXY et al. *JAMA Network Open*.

Page: implicit_page_urbanicity2

SINGLE CHOICE
In what sort of place do you currently live?

varlabel Residence

1 ○ Big city
2 ○ Smaller city
3 ○ Suburban area
4 ○ Small town
5 ○ Rural area
8 *Skipped*
9 *Not Asked*

© 2026 Wang SXY et al. *JAMA Network Open*.

Page: implicit_page_birthyr

OPEN INTEGER TEXTBOX
In what year were you born?

varlabel	Birth Year

© 2026 Wang SXY et al. *JAMA Network Open*.

Page: implicit_page_race

SINGLE CHOICE
What racial or ethnic group best describes you?

varlabel Race - US

1 ○ White
2 ○ Black or African-American
3 ○ Hispanic or Latino
4 ○ Asian or Asian-American
5 ○ Native American
8 ○ Middle Eastern
6 ○ Two or more races
7 ○ Other (open [race_other])
98 *Skipped*
99 *Not Asked*

© 2026 Wang SXY et al. *JAMA Network Open*.

Page: p_hispanic if race != 3

SINGLE CHOICE
Are you of Spanish, Latino, or Hispanic origin or descent?

varlabel Hispanic - US

1 ○ Yes
2 ○ No
8 *Skipped*
9 *Not Asked*

© 2026 Wang SXY et al. *JAMA Network Open*.

Page: gender_setup_page

SINGLE CHOICE
Are you…?

varlabel Gender

1 ○ Male
2 ○ Female
8 *Skipped*
9 *Not Asked*

© 2026 Wang SXY et al. *JAMA Network Open*.

Page: demos2

SINGLE CHOICE
What is the highest level of education you have completed?

varlabel Education

1 ○ Did not graduate from high school
2 ○ High school graduate
3 ○ Some college, but no degree (yet)
4 ○ 2-year college degree
5 ○ 4-year college degree
6 ○ Postgraduate degree (MA, MBA, MD, JD, PhD, etc.)
8 *Skipped*
9 *Not Asked*

SINGLE CHOICE
What is your marital status?

varlabel Marital Status

1 ○ Married
2 ○ Separated
3 ○ Divorced
4 ○ Widowed
5 ○ Never married
6 ○ Domestic / civil partnership
8 *Skipped*
9 *Not Asked*

SINGLE CHOICE
Are you the parent or guardian of any children under the age of 18?

varlabel Children under the age of 18

1 ○ Yes
2 ○ No
8 *Skipped*
9 *Not Asked*

© 2026 Wang SXY et al. *JAMA Network Open*.

Page: work

SINGLE CHOICE
Which of the following best describes your current employment status?

varlabel Employment Status

1 ○ Working full time now
2 ○ Working part time now
3 ○ Temporarily laid off
4 ○ Unemployed
5 ○ Retired
6 ○ Permanently disabled
7 ○ Taking care of home or family
8 ○ Student
9 ○ Other (open [employ_t]) *Not randomized*
98 *Skipped*
99 *Not Asked*

© 2026 Wang SXY et al. *JAMA Network Open*.

Page: demos5

SINGLE CHOICE
Thinking back over the last year, what was your family's annual income?

varlabel Family income

1 ○ Less than $10,000
2 ○ $10,000 - $19,999
3 ○ $20,000 - $29,999
4 ○ $30,000 - $39,999
5 ○ $40,000 - $49,999
6 ○ $50,000 - $59,999
7 ○ $60,000 - $69,999
8 ○ $70,000 - $79,999
9 ○ $80,000 - $99,999
10 ○ $100,000 - $119,999
11 ○ $120,000 - $149,999
12 ○ $150,000 - $199,999
13 ○ $200,000 - $249,999
14 ○ $250,000 - $349,999
15 ○ $350,000 - $499,999
16 ○ $500,000 or more
97 ○ Prefer not to say
998 *Skipped*
999 *Not Asked*

© 2026 Wang SXY et al. *JAMA Network Open*.

Page: inputstate_page

DROPDOWN
In which state do you live?

varlabel	State of Residence

© 2026 Wang SXY et al. *JAMA Network Open*.

- 1 Alabama
- 2 Alaska
- 4 Arizona
- 5 Arkansas
- 6 California
- 8 Colorado
- 9 Connecticut
- 10 Delaware
- 11 District of Columbia
- 12 Florida
- 13 Georgia
- 15 Hawaii
- 16 Idaho
- 17 Illinois
- 18 Indiana
- 19 Iowa
- 20 Kansas
- 21 Kentucky
- 22 Louisiana
- 23 Maine
- 24 Maryland
- 25 Massachusetts
- 26 Michigan
- 27 Minnesota
- 28 Mississippi
- 29 Missouri
- 30 Montana
- 31 Nebraska
- 32 Nevada
- 33 New Hampshire
- 34 New Jersey
- 35 New Mexico
- 36 New York
- 37 North Carolina
- 38 North Dakota
- 39 Ohio
- 40 Oklahoma
- 41 Oregon
- 42 Pennsylvania
- 44 Rhode Island
- 45 South Carolina
- 46 South Dakota
- 47 Tennessee
- 48 Texas
- 49 Utah
- 50 Vermont
- 51 Virginia
- 53 Washington

© 2026 Wang SXY et al. *JAMA Network Open*.

54 ○ West Virginia
55 ○ Wisconsin
56 ○ Wyoming
60 ○ American Samoa
64 ○ Federated States of Micronesia
66 ○ Guam
68 ○ Marshall Islands
69 ○ Northern Mariana Islands
70 ○ Palau
72 ○ Puerto Rico
74 ○ U.S. Minor Outlying Islands
78 ○ Virgin Islands
81 ○ Alberta
82 ○ British Columbia
83 ○ Manitoba
84 ○ New Brunswick
85 ○ Newfoundland
86 ○ Northwest Territories
87 ○ Nova Scotia
88 ○ Nunavut
89 ○ Ontario
90 ○ Prince Edward Island
91 ○ Quebec
92 ○ Saskatchewan
93 ○ Yukon Territory
99 ○ Not in the U.S or Canada
998 *Skipped*
999 *Not Asked*

© 2026 Wang SXY et al. *JAMA Network Open*.

Page: page_pid3

SINGLE CHOICE
Generally speaking, do you think of yourself as a ...?

varlabel 3 point party ID

1 ○ Democrat
2 ○ Republican
3 ○ Independent
4 ○ Other (open [pid3_t])
5 ○ Not sure
8 *Skipped*
9 *Not Asked*

© 2026 Wang SXY et al. *JAMA Network Open*.

Page: page_pid7

SINGLE CHOICE
$pid7text

varlabel 7 point Party ID

1	○	Strong Democrat	*Show if pid3==1*
2	○	Not very strong Democrat	*Show if pid3==1*
7	○	Strong Republican	*Show if pid3==2*
6	○	Not very strong Republican	*Show if pid3==2*
3	○	The Democratic Party	*Show if pid3 in [3,4,5]*
5	○	The Republican Party	*Show if pid3 in [3,4,5]*
4	○	Neither	*Show if pid3 in [3,4,5]*
8	○	Not sure	*Show if pid3 in [3,4,5]*
9	○	Don't know	*Show if 0*
98		*Skipped*	
99		*Not Asked*	

© 2026 Wang SXY et al. *JAMA Network Open*.

Page: voteregpage

SINGLE CHOICE
Are you registered to vote?

varlabel Voter Registration Status

1 ○ Yes
2 ○ No
3 ○ Don't know
8 *Skipped*
9 *Not Asked*

© 2026 Wang SXY et al. *JAMA Network Open*.

Page: implicit_page_presvote20post

SINGLE CHOICE
Who did you vote for in the election for President in 2020?

varlabel 2020 President Vote Post Election

1	○	Joe Biden	
2	○	Donald Trump	
3	○	Jo Jorgensen	*Not randomized*
4	○	Howie Hawkins	*Not randomized*
5	○	Other (open [presvote20post_t])	*Not randomized*
6	○	Did not vote for President	*Not randomized*
8		*Skipped*	
9		*Not Asked*	

© 2026 Wang SXY et al. *JAMA Network Open*.

Page: implicit_page_presvote24post

SINGLE CHOICE
Who did you vote for in the election for President in 2024?

varlabel 2024 President Vote Post Election

1 ○ Kamala Harris
2 ○ Donald Trump
3 ○ Robert F. Kennedy, Jr.
4 ○ Jill Stein
5 ○ Cornel West
6 ○ Chase Oliver
8 ○ Other (open [presvote24post_t])
9 ○ Did not vote for President
98 *Skipped*
99 *Not Asked*

© 2026 Wang SXY et al. *JAMA Network Open*.

Page: demos6

SINGLE CHOICE
Some people seem to follow what's going on in government and public affairs most of the time, whether there's an election going on or not. Others aren't that interested. Would you say you follow what's going on in government and public affairs ...

varlabel Political Interest

1 ○ Most of the time
2 ○ Some of the time
3 ○ Only now and then
4 ○ Hardly at all
7 ○ Don't know *Not randomized*
98 *Skipped*
99 *Not Asked*

© 2026 Wang SXY et al. *JAMA Network Open*.

Page: implicit_page_pew_bornagain

SINGLE CHOICE
Would you describe yourself as a "born-again" or evangelical Christian, or not?

varlabel Born Again (Pew version)

1 ○ Yes
2 ○ No
8 *Skipped*
9 *Not Asked*

© 2026 Wang SXY et al. *JAMA Network Open*.

Page: implicit_page_pew_religimp

SINGLE CHOICE
How important is religion in your life?

varlabel Importance of religion (Pew version)

1 ○ Very important
2 ○ Somewhat important
3 ○ Not too important
4 ○ Not at all important
8 *Skipped*
9 *Not Asked*

© 2026 Wang SXY et al. *JAMA Network Open*.

Page: implicit_page_pew_churatd

SINGLE CHOICE
Aside from weddings and funerals, how often do you attend religious services?

varlabel Church attendance (Pew version)

1 ○ More than once a week
2 ○ Once a week
3 ○ Once or twice a month
4 ○ A few times a year
5 ○ Seldom
6 ○ Never
7 ○ Don't know *Not randomized*
98 *Skipped*
99 *Not Asked*

© 2026 Wang SXY et al. *JAMA Network Open*.

Page: implicit_page_pew_prayer

SINGLE CHOICE
People practice their religion in different ways. Outside of attending religious services, how often do you pray?

varlabel Frequency of Prayer (Pew version)

1 ○ Several times a day
2 ○ Once a day
3 ○ A few times a week
4 ○ Once a week
5 ○ A few times a month
6 ○ Seldom
7 ○ Never
8 ○ Don't know
98 *Skipped*
99 *Not Asked*

© 2026 Wang SXY et al. *JAMA Network Open*.

Page: implicit_page_religpew

SINGLE CHOICE
What is your present religion, if any?

varlabel Religion

1 ○ Protestant
2 ○ Roman Catholic
3 ○ Mormon
4 ○ Eastern or Greek Orthodox
5 ○ Jewish
6 ○ Muslim
7 ○ Buddhist
8 ○ Hindu
9 ○ Atheist
10 ○ Agnostic
11 ○ Nothing in particular
12 ○ Something else (open [religpew_t])
98 *Skipped*
99 *Not Asked*

© 2026 Wang SXY et al. *JAMA Network Open*.
