# Supplementary material for: Preferences for Shared Language for Health Equity Across the Political Spectrum
Source: JAMA Netw Open. 2026 Mar 6;9(3):e260277. doi: 10.1001/jamanetworkopen.2026.0277 (PMC12966926; doi:10.1001/jamanetworkopen.2026.0277)
Supplement: Supplement 2. — Data Sharing Statement [file jamanetwopen-e260277-s002.pdf]

Data Sharing Statement

Wang. Preferences for Shared Language for Health Equity Across the Political Spectrum. *JAMA Netw Open*. Published March 06, 2026. doi:10.1001/jamanetworkopen.2026.0277

Data
Data available: No

Additional Information
Explanation for why data not available: The data that support the findings of this study are not publicly available due to privacy restrictions and the terms of participant consent but may be made available from the corresponding author upon reasonable request for research purposes.
